# Common carotid artery dissection from sportive choking

**DOI:** 10.1259/bjrcr.20230048

**Published:** 2023-10-09

**Authors:** Dr Daniel Borg, Dr Matthew Crockett

**Affiliations:** 1 Department of Neuroradiology, Beaumont Hospital, Dublin, Ireland

## Abstract

Tandem occlusions of the anterior circulation refer to the simultaneous presence of a cervical carotid artery occlusion or high-grade stenosis and an ipsilateral large vessel occlusion involving the intracranial internal carotid artery, M1 or proximal M2 middle cerebral artery. Whilst carotid occlusion usually results from progressive atherosclerotic disease, in younger individuals it may arise secondary to a dissection for which there are multiple aetiologies, with trauma being an important cause in patients having a relevant history. We present a rare case of traumatic left common carotid artery dissection in a young professional Jiu-Jitsu fighter presenting with delayed stroke symptoms and angiographic findings of a tandem occlusion. This case was successfully managed with endovascular clot retrieval and antiplatelet medication.

## Background

Sportive choking is a technique practiced in various combat sports as a fight-finishing manoeuver. The grappler compresses the neck of his opponent to restrict blood flow through the carotid arteries and jugular veins thus causing cerebral hypoperfusion. Loss of consciousness typically ensues within 10 seconds of a fighter not submitting to a choke hold.

20% of ischaemic strokes in young and middle-aged adults are related to craniocervical arterial dissection which may be spontaneous, traumatic, iatrogenic, or associated with aortic arch dissection. Traumatic dissection of the carotid artery is a very rare occurrence with a reported incidence rate of 0.08%.^1^ Several case reports in the literature highlight a potential association between cervical artery dissection (CAD) and mechanical triggers from a myriad of various sport activities, combat sports and golf being among the most prevalent offenders.^2^

Artery-to-artery embolism and cerebral hypoperfusion from progressive flow-limiting cervical artery stenosis are the two main mechanisms underlying cerebral ischaemia in patients with CAD.^3^

High clinical suspicion of this diagnosis is crucial in expediting early imaging as there is often a delay between the inciting traumatic event and onset of stroke symptoms.

## Case presentation

A healthy 5 feet 7 inch tall, right-hand dominant 46-year-old gentleman weighing 79 kg, presented to the emergency department with right-sided hemiparesis, right facial droop and aphasia, scoring 14 on the NIHSS scale. The onset of his symptoms started 6 hours after sustaining neck vasocompression from an Ezekiel choke at a Brazilian Jiu-Jitsu match the previous night. On examination, he was normotensive with a blood pressure of 130/70 mmHg and had a resting heart rate of 57 bpm. He denied any shortness of breath, had a normal respiratory rate of 16 breaths per minute and normal oxygen saturations of 98% on room air.

## Investigations

**Figure 1.**
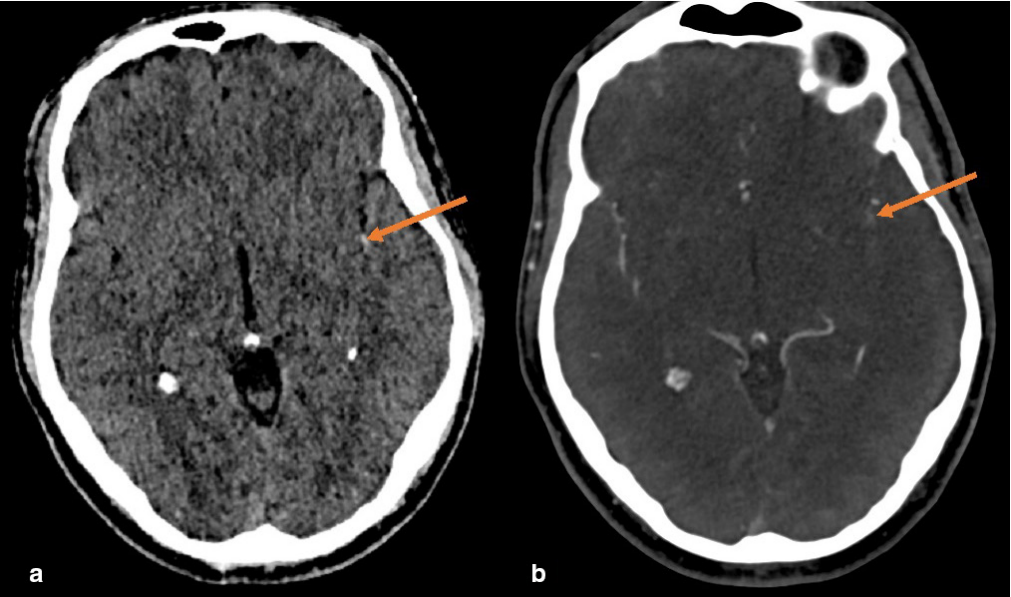
Unenhanced CT brain demonstrating hyperdense insular segment of left MCA representing hyperacute thromboembolic material within the vessel lumen (**a**). Corresponding axial intracranial CT angiogram demonstrating a proximal occlusion of the left posterior M2 MCA division (**b**). MCA, middle cerebral artery.

**Figure 2.**
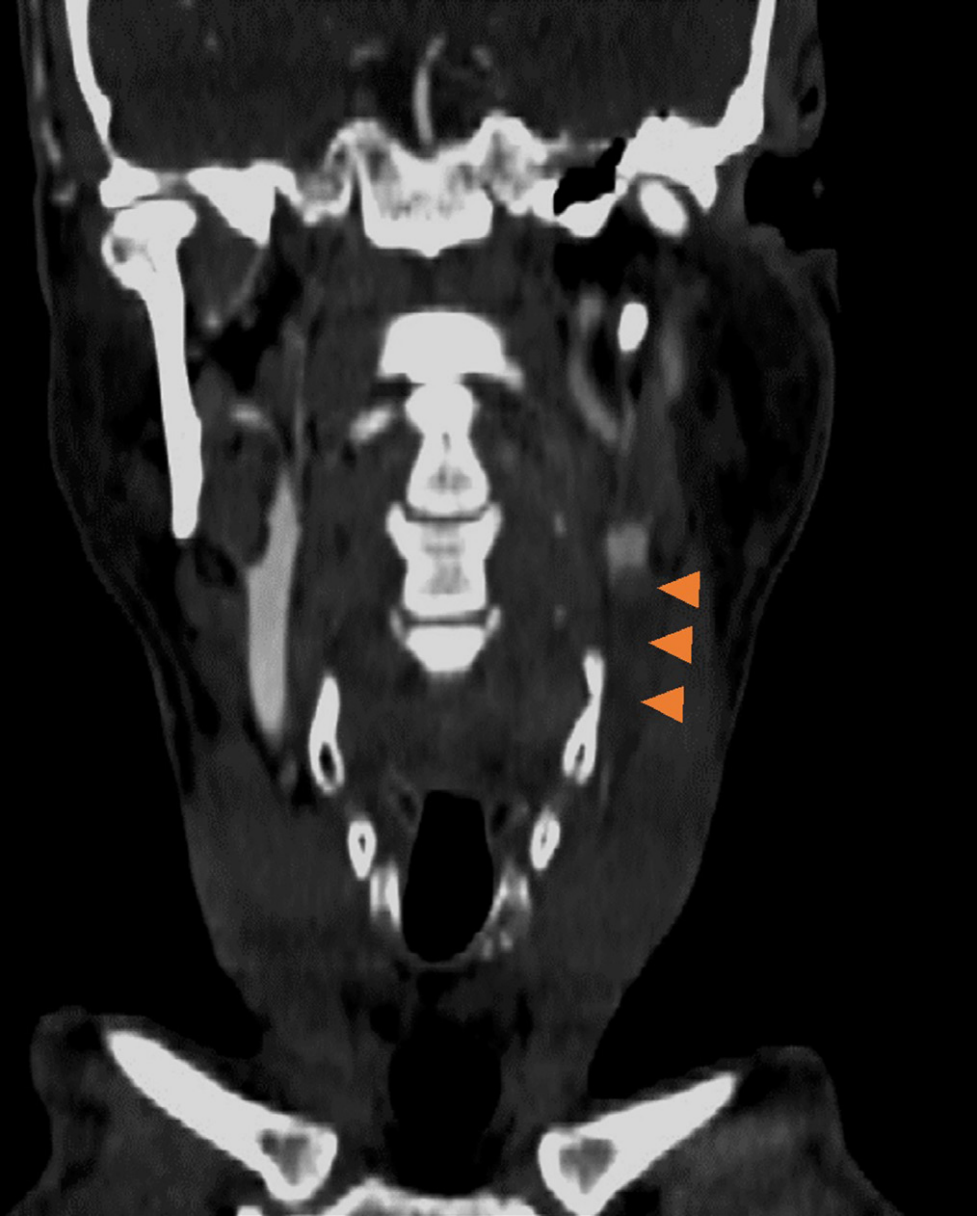
Coronal CT carotid angiogram shows absent filling of the left common carotid artery.

Patient underwent non-contrast CT brain which revealed hyperdense thrombus in the insular segment of the left middle cerebral artery (MCA) and no associated evidence of acute infarction, with an ASPECTS score of 10 (Figure 1a). Triple phase arch to vertex CT angiogram revealed complete occlusion of the left common carotid artery (Figure 2) and a tandem occlusion of a dominant proximal posterior/inferior division of the M2 MCA (Figure 1b).

The patient was thombolysed and transferred to a comprehensive stroke centre for mechanical thrombectomy.

## Treatment

**Figure 3.**
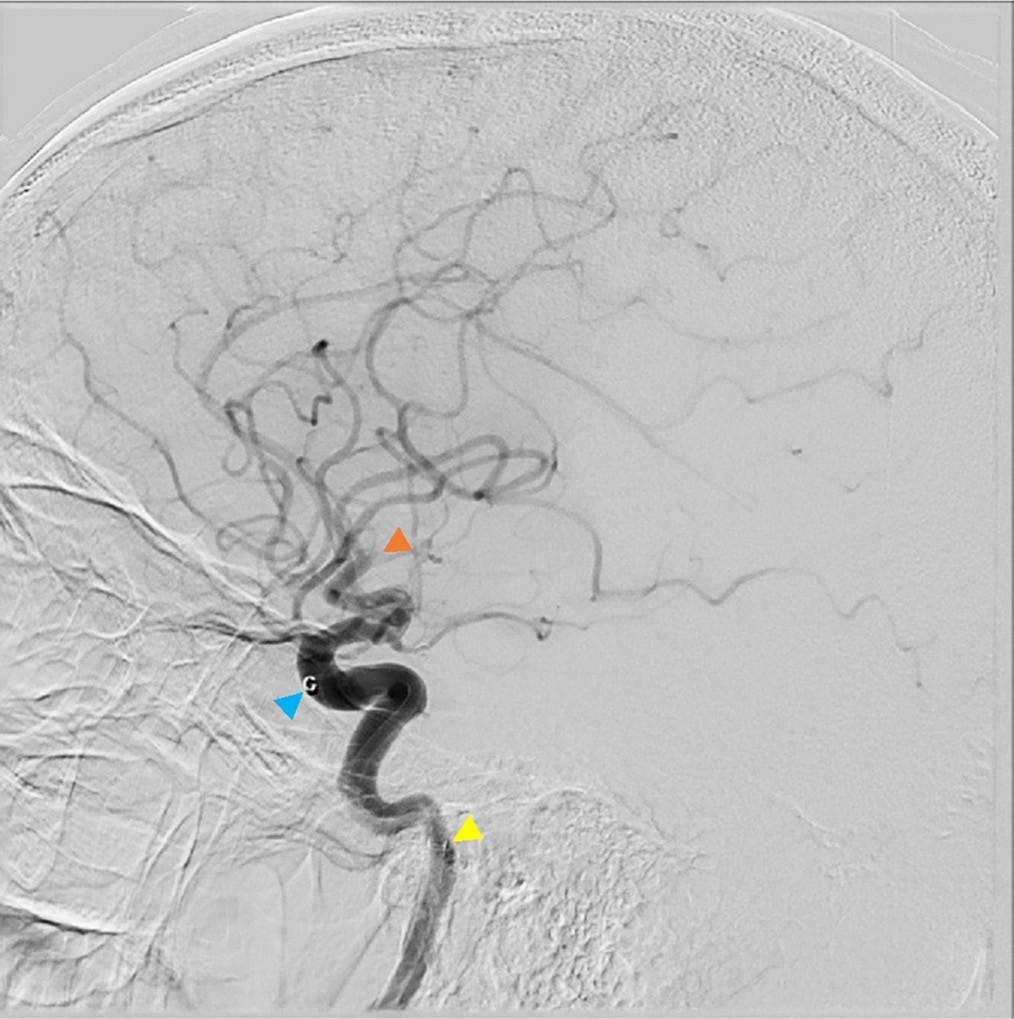
Lateral plane of intracranial left ICA angiogram pre-thrombectomy. Orange arrowhead depicts proximal M2 occlusion—TICI 2a. Aspiration catheter (blue arrowhead) just below ophthalmic artery and guide catheter (yellow arrowhead) in proximal petrous ICA. ICA, internal carotid artery.

**Figure 4.**
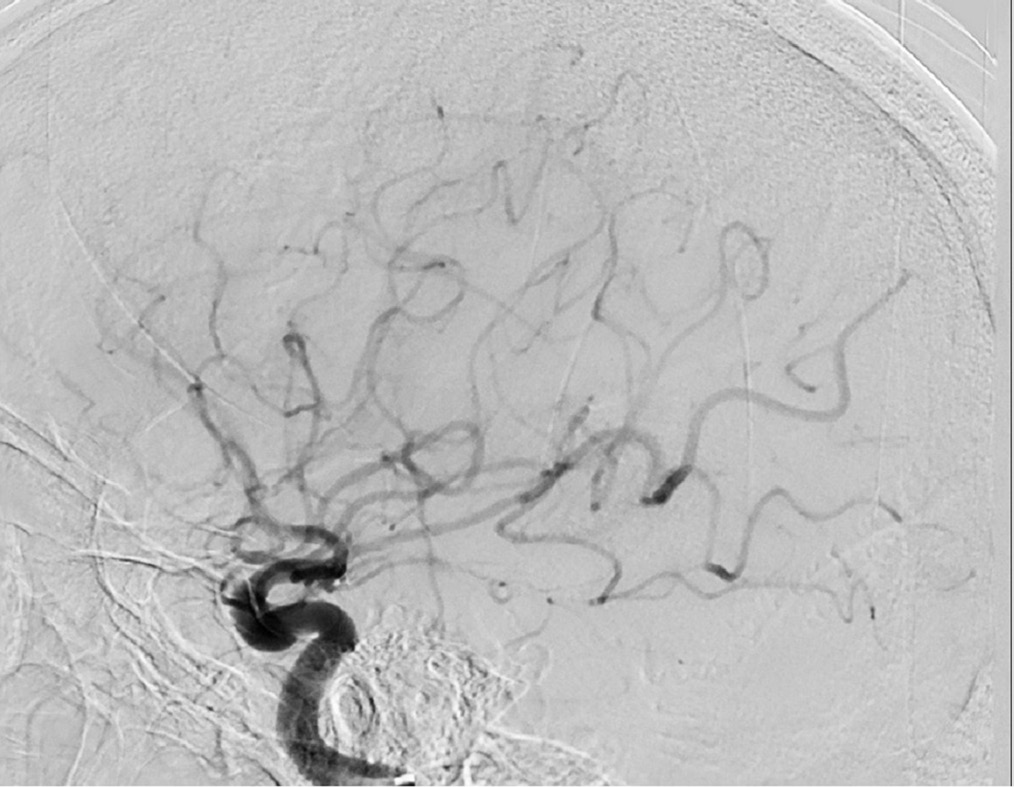
Lateral plane left ICA intracranial angiogram after single pass aspiration demonstrates complete recanalisation—TICI 3. ICA, internal carotid artery.

Local anaesthetic was administered to the right groin and an 8 French (Fr) sheath was inserted into the right common femoral artery. An 90 cm long 8 Fr Cerebase guide catheter was advanced over a 6 Fr Weinberg catheter and standard 150 cm 0.035” angled Terumo guide wire into the left proximal common carotid artery (CCA). Left carotid injection confirmed the presence of clot in the left CCA (not shown) and an associated tandem intracranial occlusion of the posterior/inferior division of the left proximal M2 MCA, with a pre-thrombectomy TICI score of 2a (Figure 3). Attempted aspiration of the left CCA clot was unsuccessful, as the distal tip of the guide catheter kept herniating out of the CCA into the Type II aortic arch. In view of this, it was decided to tackle the intracranial occlusion first. The Cerebase was negotiated into the left petrous ICA over the guide wire. A 131 cm long 6 Fr SofiaPlus aspiration catheter was next navigated to the target position at the face of the clot over a 160 cm long 0.021” Wedge microcatheter and Synchro 0.014” microwire (Wedge – M2 technique). Single pass contact aspiration resulted in complete recanalisation with a post-thrombectomy TICI score of 3 (Figure 4). A 2-point drop in NIHSS score from 14 to 12 was recorded by the stroke team immediately post-thrombectomy.

**Figure 5.**
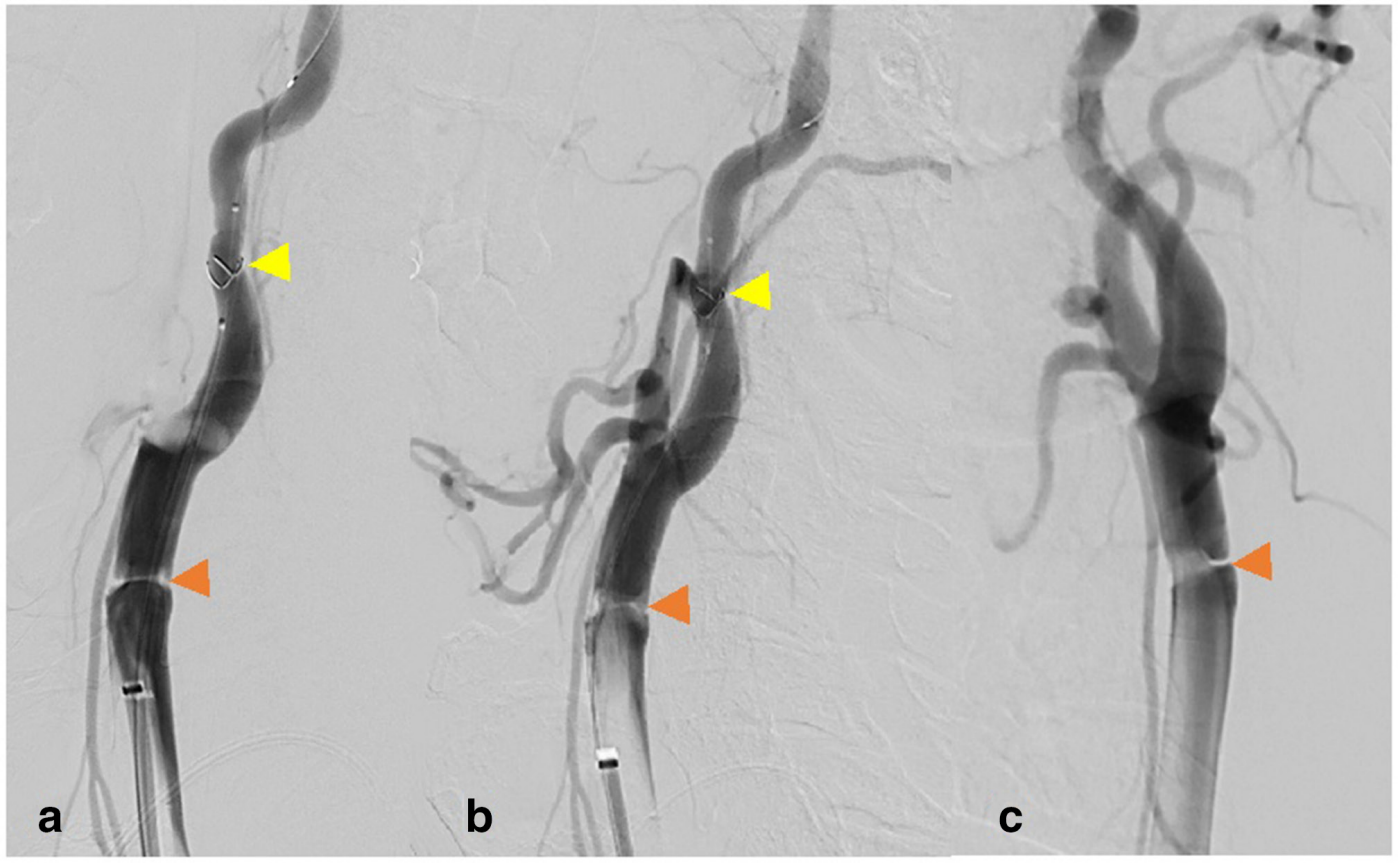
Lateral (**a, b**) and frontal (**c**) projections of extracranial left carotid angiogram pre- (**a**) and post- (**b, c**) clot retrieval from carotid bifurcation. Images a and b show Emboshield filter (yellow arrowhead) deployed in left proximal cervical ICA over delivery guide wire advanced through an 8 Fr guide catheter. Non-flow limiting dissection flap is denoted by the orange arrowhead. ICA, internal carotid artery.

Emboshield distal protection device was then deployed in left proximal cervical ICA over delivery guide wire and Cerebase was gradually retracted from ICA into proximal CCA under aspiration, successfully retrieving the clot at the carotid bifurcation. The dissection flap was non-flow limiting, hence no angioplasty or stenting was performed (Figure 5). 300 mg of intravenous aspirin was administered at the end of the procedure.

## Outcome and follow-up

**Figure 6.**
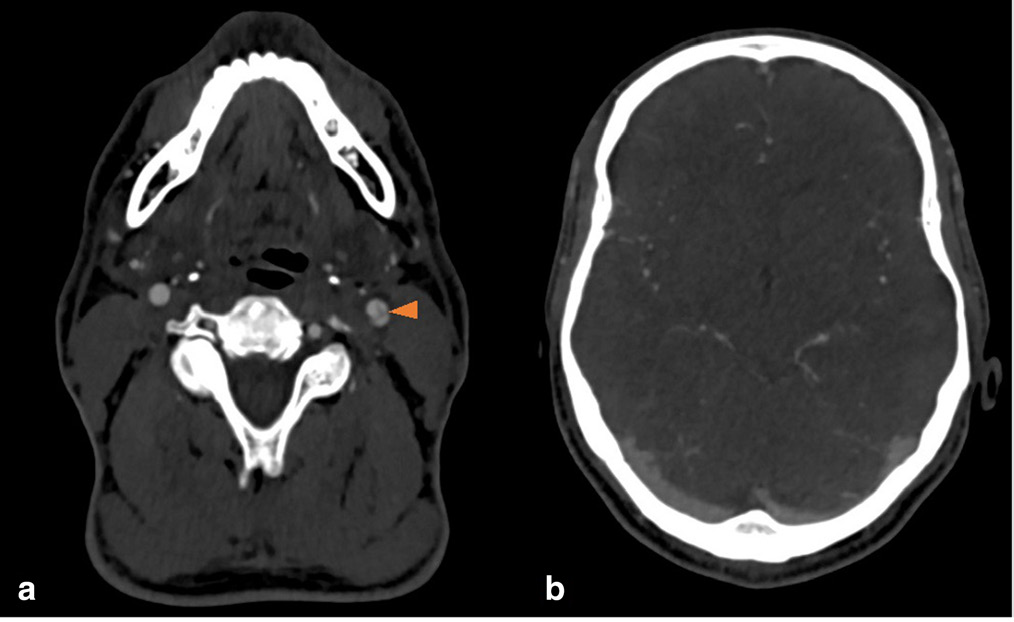
Follow-up axial CTA shows no residual clot in the left CCA and reveals the underlying intraluminal dissection flap shown by the orange arrowhead (a). Patent left MCA branches (b). CTA, CT angiography; MCA, middle cerebral artery.

Follow-up CTA 2 days following the thrombectomy showed a residual dissection flap in the left CCA but no residual clot in the CCA or M2 MCA (Figure 6).

**Figure 7.**
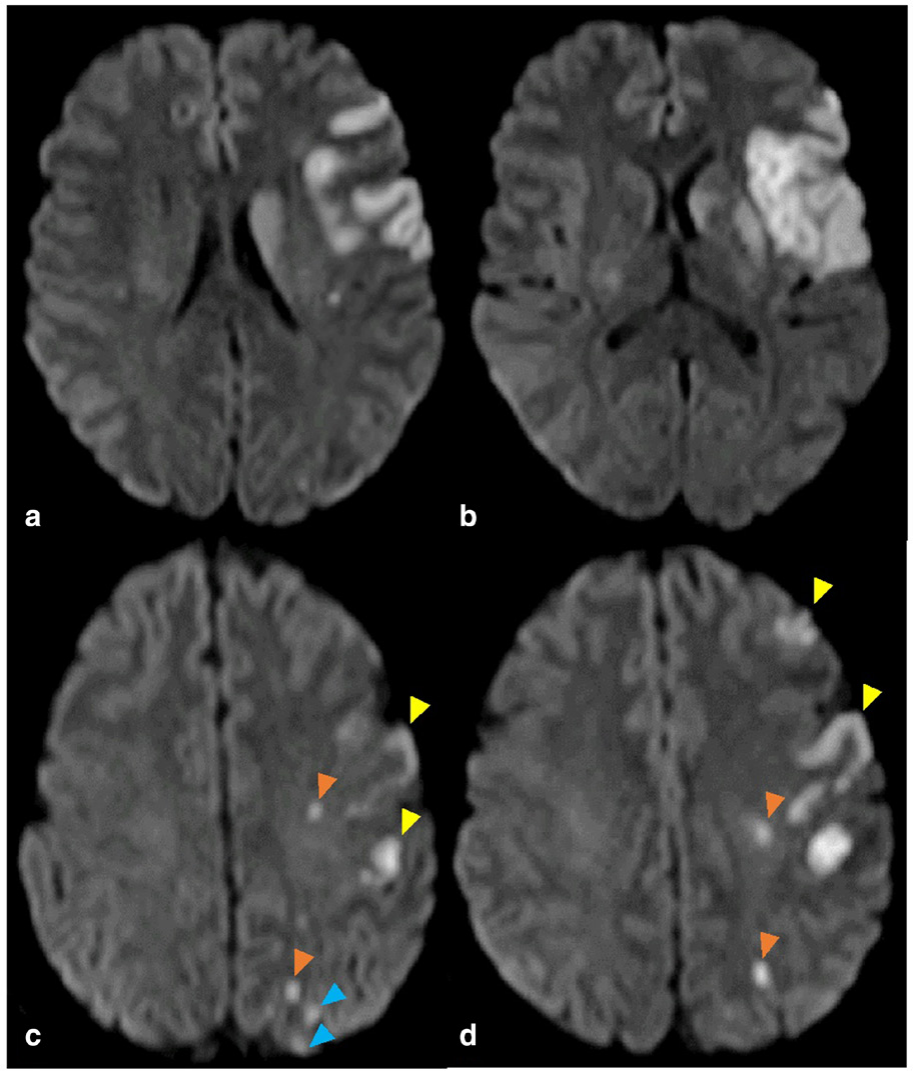
Follow-up MRI diffusion-weighted sequence. Cortical and subcortical infarct in single pial artery territory of left MCA at the ganglionic level (a, b). Embolic-type left MCA territory cortical infarcts at supraganglonic level (yellow arrowheads). Punctate infarcts in internal subcortical (orange arrowheads) and external cortical (blue arrowheads) watershed zones between left ACA/MCA vascular territories (c, d). ACA, anterior cerebral artery; MCA, middle cerebral artery.

Diffusion-weighted MRI performed 3 days post-thrombectomy showed a sizeable cortical and subcortical infarct in the left MCA territory distribution with smaller embolic-type cortical infarcts at the supraganglionic level resulting from distal MCA embolisation of fragmented thrombus. Scattered punctate infarcts were also apparent in the internal and external border zones of the left anterior cerebral artery/middle cerebral artery (ACA/MCA) and middle cerebral artery/posterior cerebral artery (MCA/PCA) vascular territories (Figure 7).

The patient was started on oral dual antiplatelet therapy consisting of Aspirin 75 mg and Clopidogrel 75 mg once daily. He was rehabilitated by the speech and language therapy team and reviewed by occupational therapy prior to discharge back to the referring hospital. During review on day 5 post-thrombectomy, patient reported complete resolution of right arm and leg symptoms but had residual motor dysgraphia and occasional dysarthria with an mRS score of 1. 90 days post-thrombectomy, patient was able to return to work and resumed training BJJ but has not competed since.

## Discussion

Brazilian Jiu-Jitsu is a type of martial art gaining increasing popularity. Various types of choke holds are practiced in this sport, exposing competitors to periods of controlled asphyxiation. The Ezekiel choke can be performed from various positions and can have several variations. When applied from a top mount position, the grappler places one arm around the back of the opponent’s neck and grabs the sleeve around his other arm which together with the weight of the inflictor’s body, presses down on the front of the opponent’s neck. This sport can be trained safely, however it may be associated with serious vascular injuries. Direct blunt force trauma to the neck may result in carotid artery injuries from various proposed mechanisms including vessel stretch due to neck hyperextension, flexion and rotation, direct vessel impact and vessel laceration from bony compression or fracture.^4,5^

Dissection of the carotid artery commonly involves the mobile segment of the vessel, 2–3 cm above the carotid bulb. At the carotid bifurcation, there is a change in the composition of the tunica media with high elastin content in the common carotid that decreases significantly in the internal carotid where smooth muscle is more abundant, a morphological feature that partially explains why the ICA is more prone to dissect.^6,7^

Radiological signs of CAD are described for various imaging modalities. Duplex ultrasound of the distal CCA, carotid bulb, extracranial cervical ICA and V2 vertebral arteries allows direct visualisation of eccentric echogenic intramural haematoma, vessel calibre change, double lumen with intimal flap, intraluminal thrombus and turbulent haemodynamic flow. Dissected ICA flow patterns vary between staccato flow with severely reduced systolic and low or absent diastolic flow, ≥50% reduction in peak systolic velocity (PSV), increased PSV or absent flow. CT angiography (CTA) best depicts pseudoaneurysms and helps distinguish the true and false lumens separated by the intimal flap. The true lumen is characteristically smaller as it is compressed by the haematoma and is contiguous with the aortic root while the false lumen is of lower contrast density due to delayed opacification. After 72 h from the inciting event, fat-saturated pre-contrast *T*_1_ weighted MRI of the neck is sensitive in the detection of the ‘crescent sign’ which infers the presence of an eccentric intramural arterial haematoma. The intra- and extracellular methaemoglobin within the haematoma in the subacute stage appears as T1 hyperintense. Contrast-enhanced MR angiography (MRA) depicts similar findings to CTA. On catheter angiography, collapse of the ICA distal to an acute stenosis is depicted by the ‘string sign’ which can be seen as a thin string of contrast within the ICA beyond the entry point of a dissection. Another appearance of dissection on digital subtraction angiography is the ‘flame sign’ which describes gradual tapering of contrast opacification and contrast pooling within the mid-cervical ICA.^8,9^

There are two postulated mechanisms of cerebral ischaemia in the setting of dissection. These are artery-to-artery embolism of intraluminal thrombus aggregating around the intimal dissection flap and haemodynamic compromise resulting from progressive flow-limiting stenosis as blood from the damaged vasa vasorum leaks into the connective tissues between the tunica intima and tunica media.^3^

The above mechanisms explain why there is often a delay between the inciting traumatic event and the onset of ischaemic symptoms. In these patients, stroke tends to happen in the first couple of days but may even occur up to 1 month after the trauma. Our patient had an MCA syndrome at presentation however, clinical signs can be transient, subtle such as headache, neck pain, mono-ocular field deficit and dizziness or even absent. This stresses the need for accurate history-taking, high clinical suspicion, and prompt investigation. Unilateral absence of a carotid pulse or a carotid bruit are also important signs to look out for in CCA occlusion/high-grade stenosis.^10^

Stroke lesions on MRI can be classified into three categories based on diffusion-weighted imaging characteristics. Pial strokes involve cortex and subcortical structures. Perforating artery strokes spare cortex but involve deep structures like basal ganglia, thalamus, internal capsule, centrum semiovale and midbrain. Junctional or border zone territory strokes involve two different arterial territories. While pial and perforating artery strokes are usually embolic in nature, watershed infarcts are more likely the result of hypoperfusion. Available literature on stroke in patients with dissection supports thromboembolism as the main contributor to ischaemia compounded by severe vessel stenosis or occlusion impeding distal flow and promoting secondary clot formation at border zones. In our patient, the DWI pattern favoured embolism as the predominant cause of the stroke lesions.^11^

Mechanical thrombectomy is an effective treatment strategy to recanalise large vessel occlusion from CAD. Unless a dissection flap is causing high-grade restriction of antegrade flow or is associated with an enlarging pseudoaneurysm at risk of rupture, there is a role for not stenting the carotid as most dissected vessels would heal spontaneously over the course of 6 months. Patients who have suffered a stroke secondary to a dissection usually have a good long-term outcome with approximately 75% regaining functional independence at 90 days and the chances of a recurrent stroke are less than 3%.^3,12^

## Learning points

- This is a case of traumatic carotid artery dissection at an atypical location within the common carotid in a young adult presenting with stroke symptoms.
- The mechanisms of cerebral ischaemia in CAD are artery-to-artery embolism of intraluminal thrombus aggregating around the intimal dissection flap and haemodynamic compromise resulting from flow-limiting stenosis.
- There is often a delay in symptom onset after the inciting traumatic event.
- Timely endovascular recanalisation and anti-thrombotic medications are complementary in the successful treatment of this entity and contribution to good long-term prognosis.
